# Next-Generation Sequencing Analysis of MiRNA Expression in Control and FSHD Myogenesis

**DOI:** 10.1371/journal.pone.0108411

**Published:** 2014-10-06

**Authors:** Veronica Colangelo, Stéphanie François, Giulia Soldà, Raffaella Picco, Francesca Roma, Enrico Ginelli, Raffaella Meneveri

**Affiliations:** 1 Department of Health Sciences, University of Milano-Bicocca, Monza, Italy; 2 Department of Medical Biotechnology and Translational Medicine, University of Milan, Milan, Italy; 3 Department of Medical and Biological Sciences, University of Udine, Udine, Italy; University of Minnesota Medical School, United States of America

## Abstract

Emerging evidence has demonstrated that miRNA sequences can regulate skeletal myogenesis by controlling the process of myoblast proliferation and differentiation. However, at present a deep analysis of miRNA expression in control and FSHD myoblasts during differentiation has not yet been derived. To close this gap, we used a next-generation sequencing (NGS) approach applied to *in vitro* myogenesis. Furthermore, to minimize sample genetic heterogeneity and muscle-type specific patterns of gene expression, miRNA profiling from NGS data was filtered with FC≥4 (log_2_FC≥2) and p-value<0.05, and its validation was derived by qRT-PCR on myoblasts from seven muscle districts. In particular, control myogenesis showed the modulation of 38 miRNAs, the majority of which (34 out 38) were up-regulated, including myomiRs (miR-1, -133a, -133b and -206). Approximately one third of the modulated miRNAs were not previously reported to be involved in muscle differentiation, and interestingly some of these (i.e. miR-874, -1290, -95 and -146a) were previously shown to regulate cell proliferation and differentiation. FSHD myogenesis evidenced a reduced number of modulated miRNAs than healthy muscle cells. The two processes shared nine miRNAs, including myomiRs, although with FC values lower in FSHD than in control cells. In addition, FSHD cells showed the modulation of six miRNAs (miR-1268, -1268b, -1908, 4258, -4508- and -4516) not evidenced in control cells and that therefore could be considered FSHD-specific, likewise three novel miRNAs that seem to be specifically expressed in FSHD myotubes. These data further clarify the impact of miRNA regulation during control myogenesis and strongly suggest that a complex dysregulation of miRNA expression characterizes FSHD, impairing two important features of myogenesis: cell cycle and muscle development. The derived miRNA profiling could represent a novel molecular signature for FSHD that includes diagnostic biomarkers and possibly therapeutic targets.

## Introduction

Facioscapulohumeral muscular dystrophy (FSHD) is the third most common myopathy, with an incidence of 1 in 14.000 in the general population. Signs of FSHD become visible in an individual's 20's (men) or 30's (women) and include loss of muscle strength in the face, shoulders, and upper arms before eventually attaining the abdomen, legs and feet. FSHD is transmitted as an autosomal dominant trait and it is thought to be mainly associated to an epigenetic alteration leading to transcriptional imbalance of the responsible genes [1], [2]. Almost all FSHD patients carry rearrangements reducing the copy number of a 3.3 kb tandemly repeated sequence (D4Z4) located at 4q35, and containing a conserved open reading frame for a double homeobox gene (DUX4). D4Z4 copy number is highly polymorphic in healthy individuals ranging between 11 and >100copies while FSHD patients carry fewer than 11 repeats [3]. Notably, although the number of D4Z4 repeats seems to be a critical determinant of the age of onset and clinical severity of FSHD, patients without D4Z4 contraction (phenotypic FSHD or FSHD2) as well as healthy individuals with D4Z4 contraction (carrier) have been also identified [4], [5]. All these observations strongly suggests that FSHD derives from the interplay of more complex genetic and epigenetic events than those already described; these additional events might take place at either 4q35 or elsewhere in the human genome.

Recently a unifying genetic model [6] that provides the expression of D4Z4 as a major cause of FSHD has been proposed. Another recent paper [7] defining the epigenetic regulation of 4q35 gene expression, demonstrated that D4Z4 deletion is associated to reduced epigenetic repression by Polycomb silencing in FSHD patients. Furthermore, *DBE-T*, a chromatin associated non-coding RNA is produced selectively in FSHD patients and it coordinates the de-repression of 4q35 genes. However, another study evaluating a large-scale population analysis of healthy and unrelated FSHD patients reports that the genetic criteria in order to manifest FSHD (D4Z4 contraction associated with a specific chromosomal background 4A-161-p(A)- pathogenic haplotype) occur in 63.7% of the analyzed FSHD patients and in 1.3% of healthy subjects [8]. Although these data certainly represent a major advance toward the definition of the molecular basis of FSHD, many questions on the disease etiology remain unexplained. Also the reported high degree of variability of the disease, in term of onset, progression and severity strongly suggests that other mechanism(s) linked to the 4q subtelomere and/or to other regions of the human genome may play a role in the disease pathogenesis.

Various recent studies have demonstrated that both FSHD myoblasts and myotubes are characterized by an extensive gene expression dysregulation mainly affecting the myogenesis and including genes linked to cell cycle control, particularly G1/S and G2/M transitions, muscle structure, mitochondrial function, oxidative stress response, and cholesterol biosynthesis [9], [10], [11].

The deciphering of the molecular basis of FSHD has been further complicated by the finding that microRNAs (miRNAs) are involved in both control and pathological myogenesis [12], [13], [14]. MiRNAs are evolutionarily conserved short non-coding RNAs (∼22 nts) that regulate the stability and/or the translational efficiency of target mRNAs. They have a very pervasive role since it is estimated that a single miRNA has the potential to regulate hundreds of target genes, and therefore, >90% of all human genes could be under miRNAs regulation [15]. MiRNAs are essential for normal mammalian development and are involved in fine-tuning of many biological processes, such as differentiation, proliferation and apoptosis [16], [17]. Emerging evidence has demonstrated that miRNA sequences can regulate skeletal myogenesis by controlling the process of myoblast proliferation and differentiation, in particular, microRNA-1, -206 and -133a/b were defined as myomiRNAs to emphasize their crucial role in myogenesis [18], [19]. More recently, a simultaneous microRNA/mRNA expression profiling of healthy myogenic cells during differentiation allowed to identify the involvement of miRNAs in the regulation of various biological processes such as cell cycle, transcription, transport, apoptosis and DNA damage [20]. Given these assumptions it was not surprising that miRNAs dysregulation was found to be involved in muscle dysfunctions [9], [12], [21].

To date, miRNA studies reported for FSHD were essentially based on the analysis of a restricted number of known miRNA sequences, thus not allowing the derivation of the full miRNA-based dysregulation network. To close this gap, here we report miRNAs expression analysis, derived by next-generation sequencing (NGS), in primary muscle cells from healthy and FSHD subjects during differentiation.

## Results

### Study design and NGS general results

**Figure 1:**
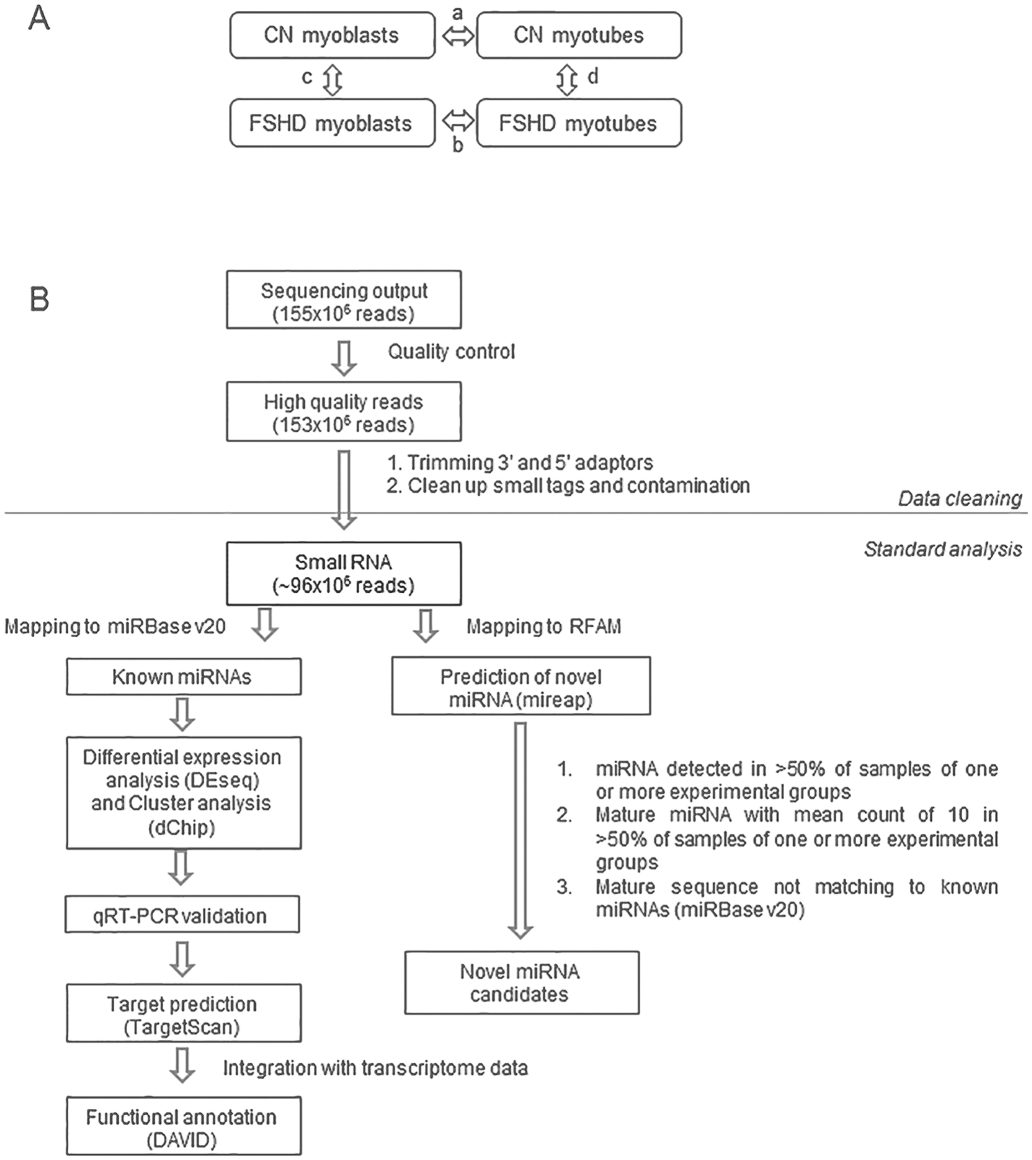
Study design and data analysis. A) Study design: Next-generation Sequencing (NGS) on three control and three FSHD myoblast cell lines before and after *in vitro* myogenic differentiation was used in order to derive miRNA modulation in: a) control myogenesis (CN myotubes vs CN myoblasts; arrow a); b) FSHD myogenesis (FSHD myotubes vs FSHD myoblasts; arrow b); c) FSHD myoblasts versus control myoblast (arrow c), and d) FSHD myotubes vs control myotubes (arrow d). B) Flow chart of filtering and analysis of NGS data. NGS generated a total of 153×10^6^ high quality reads, that were filtered for rRNA, tRNA, snRNA, snoRNA, repeat associated RNAs and intron/exon. The filtered reads (approx. 99×10^6^ reads, an average of 8×10^6^/sample) were analyzed to derive known miRNAs (R/Bioconductor) and novel miRNAs (mireap). Differentially expressed miRNAs between samples were derived by log_2_FC≧2 and p-value<0.05 parameters. The homogeneity of miRNA modulation among samples was evaluated by cluster analysis (dChip). miRNAs were then validated by qRT-PCR. Finally, target genes were predicted for modulated miRNAs and functionally annotated by DAVID.

In order to determine the entire small non coding RNAs (<35 nts) transcriptome in control (CN) and FSHD primary myoblast cell lines, before and after *in vitro* myogenic differentiation, we used next-generation sequencing (NGS). Study design was organized to allow the comparison of small non-coding RNA expression profiles between FSHD and CN myoblasts and myotubes (Fig. 1A, arrows c and d respectively) and of the two differentiation processes (Fig. 1A, arrows a and b, respectively). In order to derive biological markers (i.e. miRNA dysregulation) commonly manifested by different affected muscle districts, we used two FSHD primary myoblasts cell lines deriving from rhomboid and one from ilio-psoas muscles, and three control myoblasts from tensor fascia lata, quadriceps and vastus intermedius (Table S1).

As shown in the flow chart reported in Fig. 1B, small RNA sequencing generated a total of 153×10^6^ high quality reads. Mature miRNAs make up the majority of sequences in the 18 to 25 nts size range (65% average), with a clear peak at 22 nts in all samples. The average of known miRNAs per sample was of 556, whereas un-annotated small RNAs (new miRNA candidates) per sample were 28.

The differential expression of known miRNAs was analyzed in the different stages of muscle differentiation by DEseq analysis. Furthermore, in order to assess the robustness of our approach, some of the miRNAs identified as differentially expressed were validated by qRT-PCR using specific TaqMan miRNA assays in primary FSHD and healthy myoblasts. For these experiments we employed the same cell lines used for NGS and additional ones from different muscles, including biceps and deltoid (Table S1). As reported in Materials and Methods, the nine control and the seven FSHD cell lines showed a highly comparable extent of Desmin-positive cells and of myogenic markers modulation upon differentiation (Fig. S1). Gene targets of differentially expressed miRNAs were predicted in both control and FSHD cellular systems by using the TargetScan algorithm. Derived gene targets were filtered on two independent transcriptome profiling experiments carried out on control and FSHD myogenesis [9], [10], and shared targets were then functionally annotated by DAVID. Novel miRNAs were predicted by mireap and considered as novel candidates only if detected with a mean reads of ten in at least two out of three samples of one or more experimental groups (CN and FSHD myoblasts; CN and FSHD myotubes).

### Modulation of miRNA expression during physiological and FSHD myoblast differentiation

**Figure 2:**
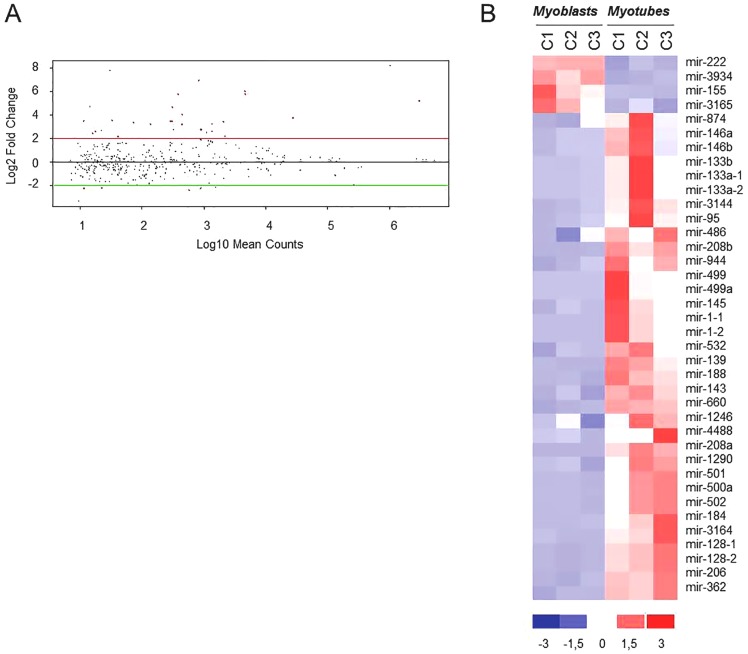
MiRNA modulation in control myogenesis. A) DEseq analysis of miRNAs differentially expressed in control myotubes vs control myoblasts (control differentiation). MiRNAs showing a modulation with log_2_FC≥2 and a p-value<0.05 are shown as red dots. B) Hierarchical clustering of the 38 modulated miRNAs (34 up-regulated and 4 down-regulated) in regard to the analyzed samples. C1:MX01010MBS; C2: MX03609MBS; C3: MX01110MBS, Control cell lines (see Table S1).

We first analyzed the data regarding physiological myogenesis (control myotubes vs control myoblasts; Fig. 1A, arrow a). Filtered miRNA reads (mapping to miRBase v20) from the three control myoblasts samples and the corresponding myotubes were analyzed for differential expression by DEseq analysis, setting the log_2_ Fold Change (log_2_FC) at ≥2 and p-value<0.05. From this analysis we evidenced that during the control myogenesis 38 miRNAs showed a modulation in their expression, and that the great majority of them (34 out of 38) were up-regulated (Fig. 2A and B).

The hierarchical clustering analysis clearly separated proliferating from differentiated cells independently of the muscle district used (tensor fascia lata, quadriceps and vastus intermedius). As expected, the muscle specific miRNAs (myomiRs) hsa-miR-1, -133a, -133b and -206, were among the most up-regulated (Fig. 2B and Table S3). Twenty-six miRNAs were already reported to be involved in muscle differentiation either in human or in mouse cells, whereas 12 miRNAs, ten up-regulated (hsa-miR-95, -146a, -874, -1246, -1290, -3164, -4488, -208a, -944 and -3144) and two down-regulated (hsa-miR-3934 and -3165), were not previously known to be involved in muscle differentiation. The full list of the miRNAs modulated during control myoblasts differentiation with corresponding FC and p-value is reported in Table S3.

**Figure 3:**
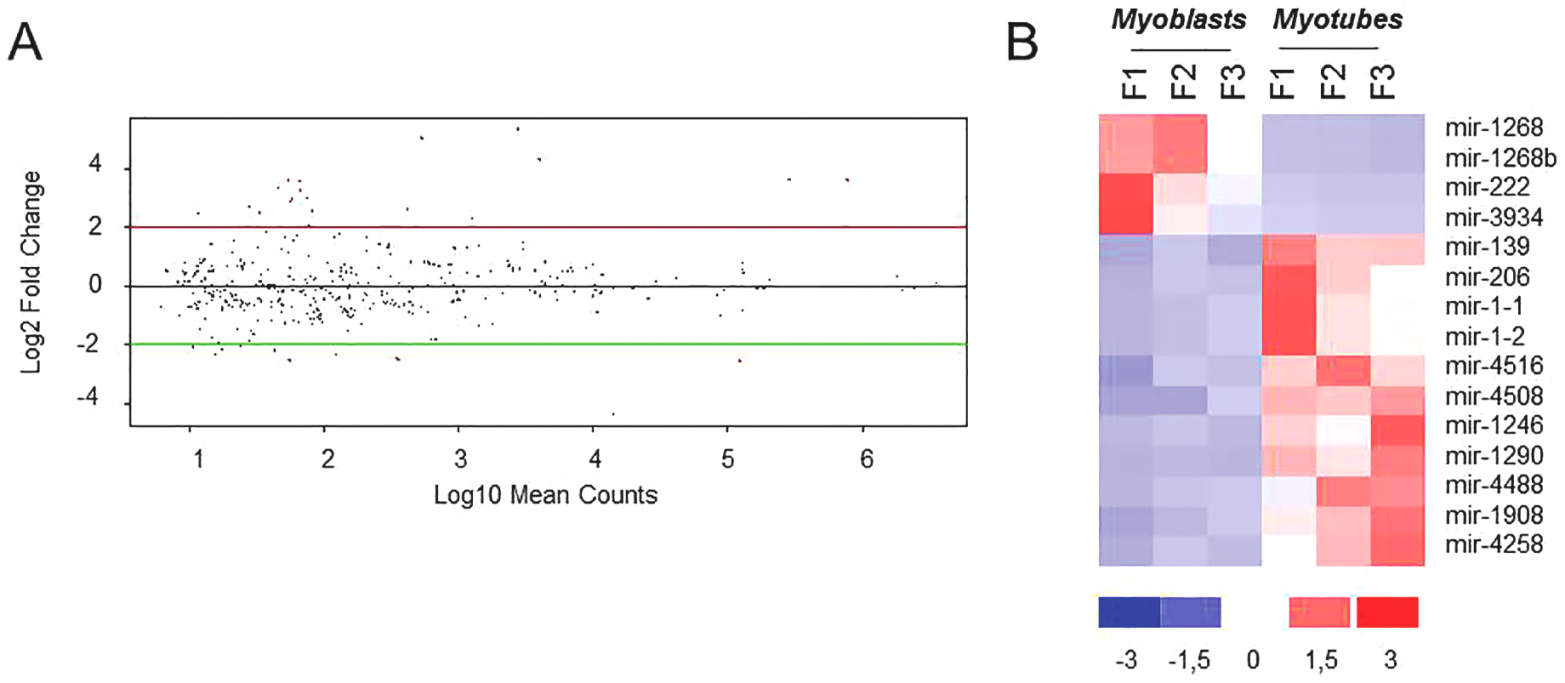
MiRNA modulation in FSHD myogenesis. A) DEseq analysis of miRNAs differentially expressed in FSHD myotubes vs FSHD myoblasts (FSHD differentiation). MiRNAs showing a modulation with log_2_FC≥2 and a p-value<0.05 are shown as red dots. B) Hierarchical clustering of the 15 modulated miRNAs (11 up-regulated and 4 down-regulated) in regard to the analyzed samples. F1:MX00409MBS; F2: MX03010MBS; F3:MX04309MBS, FSHD cell lines (see Table S1).

The same analysis was carried out on FSHD myogenesis (Fig. 1A, arrow b). As shown in Fig. 3A, the DEseq analysis evidenced the modulation of only 15 miRNAs during pathological muscle differentiation. Even in this case the hierarchical clustering analysis clearly separated proliferating from differentiated cells, independently of the muscle district (Fig. 3B). The majority of miRNAs was up-regulated (11 out of 15), including myomiR-1 and -206, although with a FC lower than that showed in control myogenesis (Table S4). MyomiR-133a and -133b showed up-regulation trend (log_2_FC>5) without reaching significance (p-value = 0.33). The full list of the miRNAs modulated in FSHD myogenesis, with corresponding FC and p-value, is reported in Table S4. Scatter plots of the reads of modulated miRNAs (for each control and FSHD proliferating and differentiated cell line) are reported in Fig. S2. To further support the results obtained by the sequencing approach, the same control and FSHD myoblast and myotube RNAs were used to analyze the expression of myomiRs (miR-1, miR-133a and miR-206) by qRT-PCR (Fig. S3). In both control and FSHD myotubes, we confirmed the general trend of myomiRs up-regulation derived by sequencing, with the pathological samples showing a lower extent of up-regulation than the normal ones.

### Dysregulation of miRNA expression in FSHD myoblasts and myotubes

**Figure 4:**
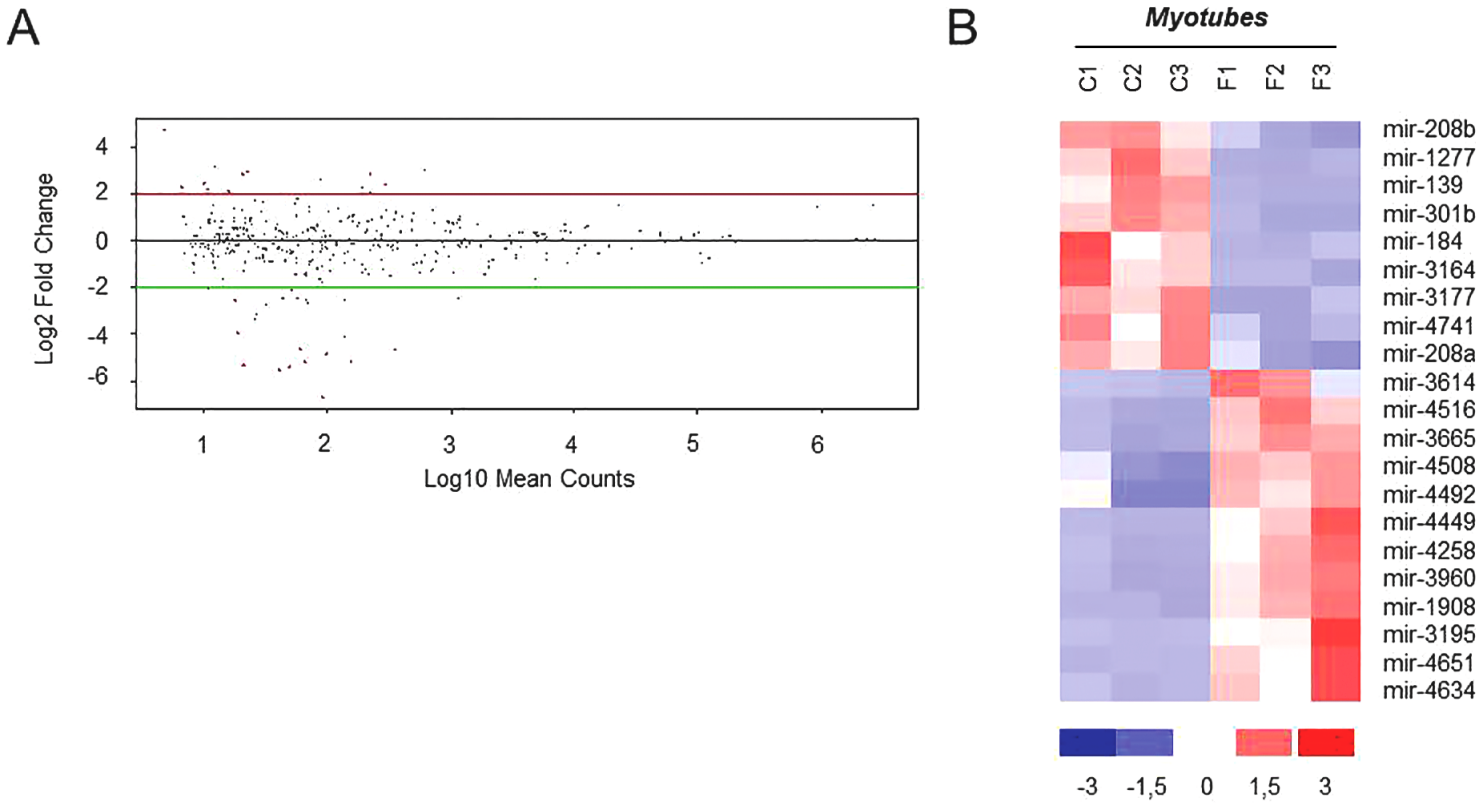
MiRNA dysregulation in FSHD myotubes. A) DEseq analysis of miRNAs differentially expressed in FSHD myotubes vs control myotubes. MiRNAs showing a differential expression of log_2_FC≥2 and a p-value<0.05 are shown as red dots. B) Hierarchical clustering of the 21 modulated miRNAs (12 up-regulated and 9 down-regulated) in regard to the analyzed samples. C1:MX01010MBS; C2: MX03609MBS; C3: MX01110MBS, Control cell lines; F1:MX00409MBS; F2: MX03010MBS; F3:MX04309MBS, FSHD cell lines (see Table S1).

We next performed DEseq analysis of miRNAs differentially expressed in FSHD myoblasts and myotubes vs controls (Fig. 1, arrows c and d). No miRNAs were found significantly dysregulated (log_2_FC≥2 and p-value<0.05) in FSHD versus control myoblasts (Fig. 1, arrows c); this result was probably due to the high variability of miRNA expression observed in myoblasts. Conversely, 21 miRNAs were found dysregulated in FSHD myotubes (Table S5 and Fig. 4A), among these 12 miRNAs were up-regulated. The hierarchical clustering analysis clearly separated the pathological samples from the control ones and the three analyzed samples of each group resulted homogeneous in miRNAs dysregulation (Fig. 4B).

### qRT-PCR Validation

**Figure 5:**
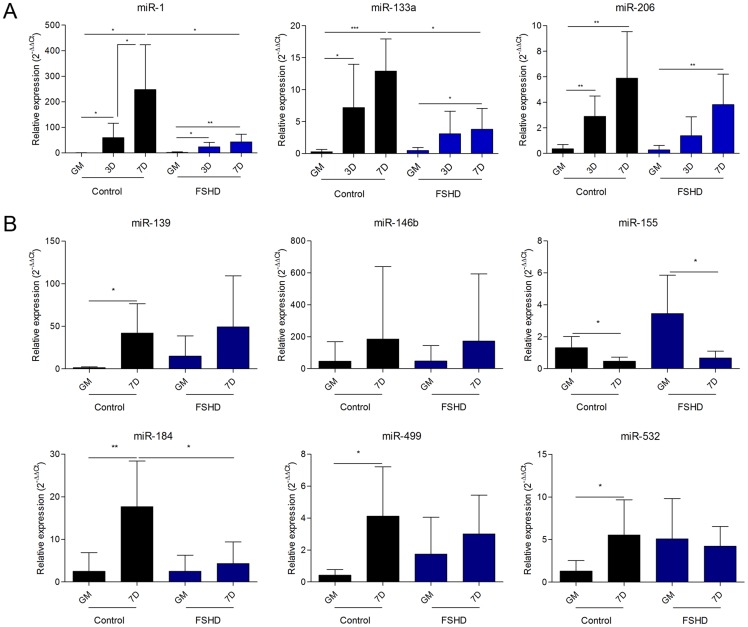
Validation of NGS data. A) qRT-PCR analysis of myomiRs (miR-1, miR-133a and miR-206) during control and FSHD myogenesis at 0, 3 and 7 days of differentiation. B) qRT-PCR analysis of six microRNAs modulated in control and/or FSHD myogenesis. GM: growth medium; 3D: 3 days of differentiation; 7D: 7 days of differentiation. * p-value<0.05; ** p-value<0.01; *** p-value<0.001.

The effective validation of deep sequencing results was performed by the TaqMan miRNA assay on all the cell lines listed in Table S1, including those already used for the NGS experiment. Particularly, for myomiR-1, -133a and -206 the assay was carried out at different time points during myogenic differentiation (0, 3 and 7 days of differentiation) (Fig. 5A). In control cells, the myomiRs progressively increased their expression with the proceeding of time of differentiation, reaching the maximum of expression at seven days, with FC values ranging from approximately 350 folds (miR-1) to 28 folds (miR-206). In FSHD cells myomiRs showed an up-regulation significantly lower than that observed in controls, reaching at day seven an expression value similar to or slightly lower than that showed by control cells at day three. Comparable fusion indexes and expression values of myogenic markers in healthy and FSHD myoblasts and myotubes (see Fig. S1) support that the obtained results are not related to a different extent of differentiation between control and pathological samples.

Six additional miRNAs were evaluated for their expression by qRT-PCR (Fig. 5B). As shown in Fig. 5 and summarized in Table 1, the qRT-PCR assays validated about the 70% of the analyzed NGS data. Particularly, the up-regulation of hsa-miR-139 and hsa-miR-146b during, respectively, FSHD and control myogenesis, and the down-regulation of hsa-miR-206 in FSHD vs CN myotubes did not reach the statistical significance showed by NGS results, while maintaining the same trend. On the contrary, the up-regulation of miR-133a in FSHD myogenesis, the down-regulation of hsa-miR-1 and hsa-miR-133a in FSHD vs CN myotubes, and the down-regulation of hsa-miR-155 in FSHD myogenesis already observed in the NGS analysis became significant in the qRT-PCR analysis.

**Table 1:** qRT-PCR validation of NGS data.

miRNA	Control myogenesis				FSHD myogenesis				FSHD vs control myotubes			
	Deep seq		qRT-PCR		Deep seq.		qRT-PCR		Deep seq		qRT-PCR	
	FC	p-value	FC	p-value	FC	p-value	FC	p-value	FC	p-value	FC	p-value
*miR-1*	293.3	2E-05	352.2	0.007	12.4	3E-06	25.8	0.007	−2.8	0.510*	−5.7	0.033
*miR-133a*	64.5	2E-05	44.8	1E-04	40.7	0.335	7.9	0.03	−1.2	0.978*	−3.4	0.007
*miR-139*	24.7	4E-07	28.9	0.018	5.7	0.021	3.3	0.189*	−7.3	0.002	1.2	0.905*
*miR-146b*	5.5	0.016	3.9	0.419*	2.2	0.858	3.6	0.460	4	0.510	−1.1	0.955
*miR-155*	−5.6	0.003	−2.8	0.031	−1.4	0.869*	−5.2	0.019	2.7	0.263	1.4	0.413
*miR-184*	53.9	9E-11	7.1	0.003	7.4	0.145	1.7	0.493	−5.3	0.002	−4.1	0.035
*miR-206*	36.1	4E-17	28.7	0.009	12.03	1E-06	11.8	0.008	−2.9	0.002	−3.1	0.07*
*miR-499a*	122.1	0.005	9.7	0.031	5.9	0.143	1.7	0.359	−8.3	0.338	−1.4	0.525
*miR-532*	9.3	3E-06	4.3	0.037	3.9	0.131	1.2	0.699	−1.99	0.456	−1.3	0.511

Fold Change and p-value of nine miRNAs derived by deep sequencing and subsequently analyzed by qRT-PCR. Asterisked values refer to miRNAs that did not reach significance, although showing the same trend of variation in both analyses.

### Comparison of FSHD and control myogenesis

**Figure 6:**
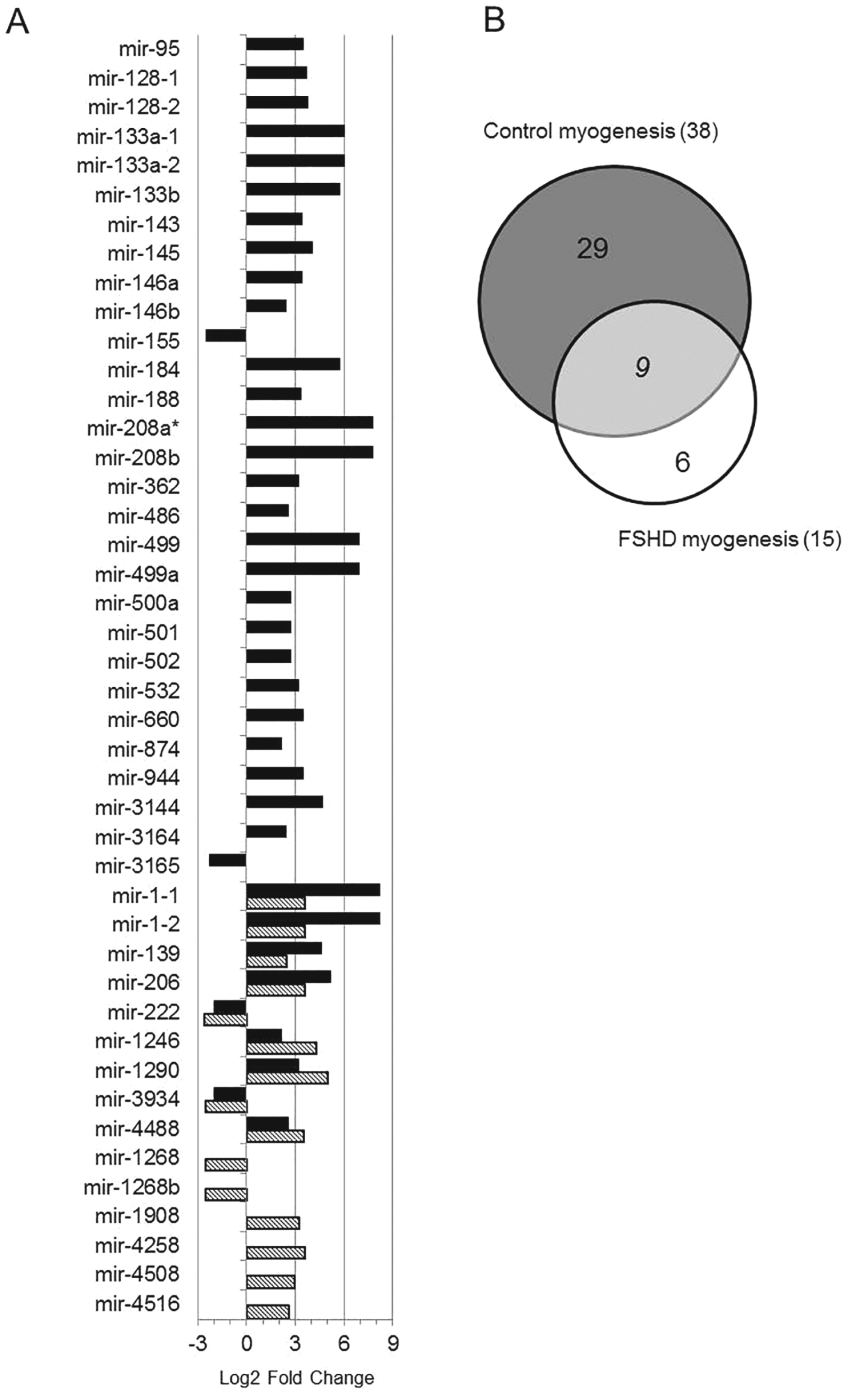
Comparison of miRNA modulation in control and FSHD myogenesis. A) Black and striped bars identify the Fold Change of miRNAs modulated respectively, in control and FSHD myogenesis. Bars on the left and on the right represent, respectively, down- and up-regulated miRNAs. *hsa-mir-208a showed infinite FC value (see Table S3). B) Venn diagram showing the number of miRNAs unique to FSHD (white) or control (grey), and shared (light grey) by FSHD and control differentiation processes.

The comparison of miRNA modulation between control and FSHD differentiation processes is reported in Fig. 6A, where black and striped bars identify the Fold Change of miRNAs up- and down-regulated, respectively, in control and FSHD myogenesis. From this comparison it was possible to derive that FSHD differentiation lacks the modulation of 29 miRNAs, the majority of which (27/29) was up-regulated in control differentiation (black bars in Fig. 6A, and Fig. 6B); while six miRNAs (4 up- and 2 down-regulated) were modulated only during the FSHD differentiation process (striped bars in Fig. 6A and Fig. 6B). Nine miRNAs showed the same trend in both processes (Fig. 6A and B), but with differences in Fold Change values. Among these, miRNAs pivotal for the myogenic process, such as hsa-miR-1, -206 and -222, were included. Thus, FSHD myogenesis differs from control myogenesis for the complete (35) or partial (9) dysregulation of a total of 44 miRNAs.

### Prediction of miRNA target genes

To understand the functional impact of miRNA dysregulation during FSHD myogenesis we used TargetScan prediction software to derive potentially affected targets. In order to improve target prediction accuracy, a common approach is to combine the output of two or more prediction algorithms, however this strategy has been proved inefficient [20]. Therefore, we have used a single algorithm, TargetScan, which uses many parameters to predict target scoring without omitting miRNAs with multiple target sites [22]. Since the binding of a miRNA to the 3′ UTR of its mRNA target predominantly act to decrease target mRNA levels [23] we decide to essentially focalize our attention on mRNA targets showing an opposite expression value compared to the analyzed miRNA. Normally, this approach has been carried out on mRNA expression profile derived by using the same cells from which the miRNA expression profile has been derived [11], [20], [21]. However, the comparison of mRNA expression profiles derived by myoblast cell lines or biopsies from different FSHD patients and controls clearly evidenced a certain variability in the obtained results [5], [9], [10], [11], [24], [25]. In addition, mRNA expression differences were also found by analyzing different muscles, such as biceps and deltoids [11]. To reduce sample variability, we filtered the predicted mRNA targets on two chip expression data (GSE26061 [9]; GSE26145 [10]), sharing *in vitro* myogenic differentiation protocol and platform although using primary FSHD and control cell lines different from those analyzed in this work. Functional classes corresponding to the filtered mRNAs were assigned by DAVID Gene Ontology Database (Table 2). As shown in Fig. 6, control myogenesis showed the modulation of 38 miRNAs (4 down- and 34 up-regulated), whereas FSHD myogenesis was characterized by 15 dysregulated miRNAs (4 down- and 11 up-regulated) and the lack of modulation of 29 miRNAs. Applying the rationale described above, we derived a total of 139 and 78 down- and up-modulated mRNAs in control myogenesis (potentially “validated” target, Table S6), and a total of 37 down- and 18 up-regulated transcripts in FSHD myogenesis (potentially “validated” target, Table S7). In control myogenic differentiation, the majority of down-regulated genes belonged to cell cycle (27 entries), DNA metabolic process (17 entries), cytoskeleton organization (11 entries), angiogenesis (8 entries) and signal transduction (19 entries); genes involved in cell adhesion (9 entries), regulation of cell migration (5 entries), muscle development (7 entries), lipid biosynthetic process (6 entries) and response to insulin (4 entries) were found up-regulated (Table 2). Conversely, in FSHD myogenesis genes belonging to muscle development (3 entries) and cell adhesion (5 entries) were down-regulated, whereas those involved in regulation of signal transduction (3 entries) were up-regulated. All the identified biological processes, except the down-regulation of cell adhesion in FSHD samples, showed a significant p-value ranging from 3.4E-10 to 3.2E-02 (see Table 2). It is noteworthy that target genes involved in two important biological processes of myogenesis (i.e. cell cycle and striated muscle development) subjected to miRNA control were, as expected, down- and up-regulated, respectively, in control cells. In FSHD myogenesis, on the contrary, the cell cycle was not down-regulated, and control of striated muscle development was down-regulated. It is important to notice that this analysis did not take into account the different FC showed by the nine miRNAs shared by control and FSHD myogenesis.

**Table 2:** Functional classification of predicted target genes in control and FSHD myogenesis.

Biological processes	CONTROL MYOGENESIS		FSHD MYOGENESIS	
	*Down (p-value)*	*Up (p-value)*	*Down (p-value)*	*Up (p-value)*
*Cell cycle*	+(3.4E-10)			
*DNA metabolic process*	+(2.8E-06)			
*Cytoskeleton organization*	+(2.7E-03)			
*Angiogenesis*	+(1.2E-03)			
*Signal transduction*	+(8.2E-03)			+(3.2E-02)
*Cell migration*		+(6.0E-03)		
*Cell adhesion*		+(1.2E-02)	+(7.4E-02)	
*Striated muscle development*		+(1.6E-02)	+(5.8E-03)	
*Sterol biosynthetic process*		+(1.0E-02)		+(5.0E-04)
*Response to insulin*		+(3.4E-03)		

Functional classification of predicted target genes of modulated miRNAs in control and FSHD myogenesis, filtered on GSE26061 [9] and GSE26145 [10]. For full lists of considered miRNAs and predicted target genes refer to Tables S3, S4 and S6, S7, respectively.

### Identification of novel miRNAs

To identify novel potential miRNAs involved in human muscle system, the unclassified tags were further processed by mireap (http://sourceforge.net/projects/mireap). We considered only tags meeting the default parameters, expressed in all experimental groups or preferentially expressed in one or more sample groups (i.e. proliferating vs differentiated cells, or FSHD vs control cells) and with mean read counts per group greater than ten. By using these criteria we identified a total of 13 novel candidate miRNA genes. In Table S8 are reported the main features of these novel miRNA genes, including chromosome location and genomic organization, mfe (minimum free energy), sequence and structure of hairpin precursor, and sequence of 5p or 3p. A summary of these data is reported in Table 3: six miRNAs showed a preferential expression in myoblasts (both in FSHD and control) and four miRNAs seemed to be specific for myotubes. The remaining three miRNAs characterized all the considered groups (both control and FSHD myoblasts and myotubes). Among the 13 novel miRNAs, two miRNAs (namely hsa-miR-m1-3p and hsa-miR-m13-5p) had already been detected by analyzing prostate and breast tumor cells [26], [27] and the mature hsa-mir-m9-3p showed 100% sequence similarity with hsa-mir-574 whose gene however differs in genomic location [28].

**Table 3:** Novel miRNAs predicted by mireap.

Name	Chromosome location	Mature miRNA sequence	Length	Genomic context	Expression n.samples	Other evidence
*hsa-miR-m1-3p*	chr11:122022800–122022877	AAAAGGGGGCTGAGGTGGAGG	21	intronic	12/12 (higher expression in myoblasts)	PMID:21346806
*hsa-miR-m2-3p*	chr11:125757935–125758025	AGGGGCGCGGCCCAGGAGCTCAGA	24	intronic	5/6 myoblasts	no
*hsa-miR-m3-3p*	chr13:111102986–111103008	AGCTGGGGATGGAAGCTGAAGCC	23	intronic	4/6 myoblasts	no
*hsa-miR-m4-5p*	chr14:74998697–74998789	CTGCTCTGATGTCTGGCTGAGC	22	intronic	5/6 myoblasts	No
*hsa-miR-m5-5p*	chr15:41592311–41592403	ATCATTTGGCAGGGGGTAGAGTA	23	intergenic	3/3 FSHD myotubes	No
*hsa-miR-m6-3p*	chr15:45493361–45493452	TTGTGGAAACAATGGTACGGCA	22	overlaps repeat/tRNA	4/6 myotubes	No
*hsa-miR-m7-5p*	chr17:8042708–8042779	GAGTTAGCGGGGAGTGATATATT	23	overlaps repeat/tRNA	4/6 myoblasts	No
*hsa-miR-m8-3p*	chr:6:28918819–28918903	TCGGGCGGGAGTGGTGGCTTTT	22	overlaps repeat/tRNA	12/12	No
*hsa-miR-m9-3p*	chr8:79679467–79679541	TGAGTGTGTGTGTGTGAGTGTGA	23	intronic	9/12 (all groups)	mature miRNA identical to hsa-mir-574, different genomic location PMID:17604727
*hsa-miR-m10-5p*	chrX:18651329–18651427	AACTTTGGAATGTGGTAGGGTA	22	intronic	3/3 FSHD myotubes	No
*hsa-miR-m11-5p*	chrX:40478974–40479066	ATCATTTGGCAGGGGGTAGAGTA	23	intergenic	3/3 FSHD myotubes	No
*hsa-miR-m12-3p*	chr13:111102941–111103018	AGCTGGGGATGGAAGCTGAAGCC	23	intronic	4/6 myoblasts	No
*hsa-miR-m13-5p*	chr20:3194751–3194835	CAAAATGATGAGGTACCTGATA	22	Intronic	6/6 myoblasts	PMID:21152091

Furthermore, it is interesting to note that no sample showed reads generated from the D4Z4 region. This observation, derived either by the analysis of the filtered out repeats or by the re-mapping NGS raw data to specific D4Z4-bearing chromosome regions such as 4q and 10q, suggests that short transcribed sequences from D4Z4 array may have a length greater than 35 nts, the threshold used to build our libraries.

## Discussion

The paper reports the first complete analysis of miRNA modulation during *in vitro* differentiation in both control and FSHD-derived myogenic cells. Myogenesis is a complex process that includes proliferation, differentiation, and formation of myotubes and myofibers. These molecular events are regulated by myogenic factors and miRNAs. MiRNAs specifically expressed in skeletal and cardiac muscles are called myomiRs, to imply their important roles in the regulation of muscle development and differentiation [13], [19], [29]. Recently miRNA dysregulation has been reported in FSHD [9], [12], [21]. However, due to the approaches used, these studies were limited for the number and type of miRNAs that could be simultaneously investigated; in addition they would not detect miRNAs expressed at low level and excluded discovery of novel miRNAs. Thus, to get the whole pattern of miRNA dysregulation in FSHD we used a next-generation sequencing (NGS) approach. Previous work aimed at identifying biomarkers in FSHD by the transcriptional profiling found muscle-type specific patterns of gene expression [11]. Similarly, DUX4-fl expression was found to vary between myotubes derived from different muscle groups [30]. Therefore, we tailored the experimental protocol to derive FSHD and control miRNA profiles common to different muscles. To this aim, due to inter-individual genetic heterogeneity, from deep sequencing data we considered only miRNA modulation with FC≥4 (log_2_FC≥2) and p-value<0.05. Then the derived miRNA expression in both FSHD and control myogenesis was validated by qRT-PCR in all the available FSHD and control cell lines.

Control myogenesis showed the modulation of 38 miRNAs, the majority of which (34 out 38) were up-regulated. The up-regulated miRNAs included those previously identified as key regulators of both proliferation and differentiation of myogenic cells and for this reason called myomiRs: hsa-miR-1, -133a, -133b and -206 [19], [31], [32]. The obtained results are in agreement but also expand what is known about miRNA modulation during *in vitro* human myogenic differentiation. Among the modulated miRNAs, 27 were in fact already reported to be involved in muscle differentiation either in human or in mouse cells [20], [33]. Conversely, 12 miRNAs, ten up-regulated (hsa-miR-95, -146a, -874, -1246, -1290, -3164, -4488, -208a, -944 and -3144) and two down-regulated (hsa-miR-3934 and -3165), were not previously detected to be differentially expressed during control myogenesis. In comparison with a previous work [20], the reduced number of modulated miRNAs during control myogenesis that we derived is probably due to the choice of higher FC value (FC≥4). Furthermore, the observed up-regulation of myomiRs strongly supports the validity of used cell lines and differentiation protocol. Interestingly, some up-regulated miRNAs not previously reported to be involved in muscle differentiation, were previously shown to affect cell proliferation by targeting HDAC1 (hsa-miR-874), impairing cytokinesis (hsa-miR-1290), inhibiting cell growth (hsa-miR-95) and regulating differentiation of smooth muscle cells (hsa-miR-146a) [34], [35], [36], [37].

Control myogenesis also showed the possible involvement of some of the novel miRNAs we derived by NGS. In this regard, six out of the 13 identified novel miRNAs (see Table 3) seem to characterize the proliferating status of muscle cells (myoblasts, miR-m2-3p, -m3-3p, -m4-5p, -m7-5p, -m12-3p, and –m13-5p) and one the differentiated status (myotubes, miR-m6-3p). Two, hsa-miR-m1-3p, and hsa-miR-m13-5p, have been previously identified by the NGS approach and validated in breast and prostate cancer cells (identified respectively as hsa-miR-B19 and hsa-novel-miR-08) [26], [27]. Further experiments are thus necessary to validate and determine the possible involvement in muscle cells differentiation of these novel miRNAs.

The comparison of control and FSHD myogenesis clearly evidenced a reduced number of modulated miRNAs in FSHD than in control muscle cells, thus suggesting that a complex dysregulation of miRNA expression characterizes the dystrophy. In total, nine miRNAs were shared between the two processes and these included myomiR-1 and -206, with FC values of up-regulation during differentiation lower than those derived for control cells. Moreover, qRT-PCR analysis proved that in control cells the up-regulation of myomiRs is higher than in FSHD ones by a FC ranging from 2.4–5.5× for hsa-miR-206 and 133a to 13× for hsa-miR-1. Furthermore, the kinetic of myomiRs up-regulation during FSHD myogenesis strongly suggests a defect in late stages of the differentiation process. Other differences between control and FSHD differentiation are represented by six miRNAs (i.e. hsa-miR-1268, -1268b, -1908, 4258, -4508- and -4516) not modulated in control cells and that therefore could be considered FSHD-specific, likewise three novel miRNAs (hsa-miR-m5-5p, hsa-miR-m10-5p and hsa-miR-m11-5p) that seem to be specifically expressed in FSHD myotubes (see Table 3). Of interest, hsa-miR-1268 exhibited a significant differential expression during the differentiation of pluripotent human embryonic stem cells into embryoid bodies [38]. In summary, FSHD myogenesis differed from control myogenesis by the loss of modulation of 29 miRNAs (black bars in Fig. 6A, and Fig. 6B) and the acquisition of modulation of six miRNAs, two down-regulated and four up-regulated (striped bars in Fig. 6A, and Fig. 6B). Among the nine miRNAs shared by the two differentiation processes (black and striped double bars in Fig. 6A), the myomiRs showed a significant deficit of expression in late phases of FSHD differentiation. Moreover, the comparison of miRNA expression between control and FSHD myoblasts or myotubes detected 21 dysregulated miRNAs only in myotubes (12 up-regulated and 9 down-regulated). The lack of differentially expressed miRNAs in FSHD myoblasts may be explained both by a high variance of miRNA expression showed by myoblasts and by the high FC used.

Some discrepancies between the data we derived and those recently reported in a similar cellular system [21] require several considerations. First, the methodological approach (NGS against transcriptome profiling), and consequently the cut-off used make the results obtained not comparable; second, both healthy and FSHD myoblast cell lines characterized by a high percentage of DES+ cells were induced to differentiate for three days [20] and for seven days (herein). Lastly, our study design was set up in order to derive a FSHD miRNA profiling possibly shared by different muscle districts and including all the microRNAs present in miRBase (release 20), as well as novel miRNAs. In this regard, it is noteworthy that if we had used the microRNA panel version 1.0 (a TaqMan low density array containing 365 miRNAs) instead of the NGS approach, we would have only detected the modulation of five miRNAs during differentiation of FSHD myoblasts (namely hsa-miR-1-1, 1-2, -206, -222 and -139), instead of the fifteen effectively found (see Table S4). Thus, as previously shown in other cellular systems [26], [27], [39], [40] the deep sequencing approach allowed us to derive a more complete view of miRNA dysregulation in FSHD.

Our data strongly suggest that, in addition to the recently reported up-regulation in proliferating FSHD vs control cells, which however did not result in a complete down-regulation of the corresponding target genes [21], a defect of myomiRs expression also characterize late stages of FSHD differentiation. In fact, the extent of myomiRs expression in FSHD cells after seven days of differentiation was similar to or lower than that found at three days in control cells. Thus, besides the reported up-regulation of myomiRs in FSHD myoblasts due to the early euchromatization of their promoters [21] other defects could be responsible of their down-regulation during late stages of differentiation. In this regard it is possible to hypothesize a defect in FSHD myotubes at the myomiRs transcriptional or post-transcriptional levels, such as a decrease of myogenic differentiation factors controlling their transcription (i.e. *MEF2*) [41], or of factors controlling their processing. The latter hypothesis agrees with previous results showing that FSHD myotubes are characterized by the down-regulation of a gene (*Dicer1*) controlling the cytoplasmic maturation of pre-miRNAs [10].

Our data allowed us to confirm a few miRNAs previously found dysregulated by independent analysis of ten major skeletal muscle disorders, including FSHD [12], [42]. Among the miRNAs we derived to be deregulated during FSHD muscle differentiation, four miRNAs (miR-146a, -146b, -155, -222) were consistently found up-regulated in six or more muscular disorders, including FSHD, whereas miR-501 was found dysregulated in five muscle diseases, but not in FSHD. Furthermore miRNA-486, a muscle enriched miRNA, previously found significantly reduced in patients with DMD [12], was found up-regulated in the present study. Interestingly overexpression of this miRNA in mouse primary myoblasts resulted in increased proliferation and thus in altered cell-cycle kinetics [43].

In order to understand the functional outcome of miRNA dysregulation in FSHD, the derived up- and down-regulated target genes were functionally clustered into biological processes. This approach when applied to healthy muscle differentiation evidenced two important features of myogenesis: cell cycle and muscle development. Effectively, as muscle differentiation proceeds, sustained by the up-regulation of myogenic markers (due to the down-regulation of the corresponding miRNA regulators), the cell proliferation program must slow down due to the up-regulation of miRNA controlling genes involved in this process. An opposite trend of the two biological processes was found to characterize FSHD myogenesis. In fact, down-regulated genes were essentially involved in the regulation of striated muscle tissue development, and no regulation of cell cycle was observed. Thus in FSHD cells miRNA dysregulation affects two important aspects of differentiation leading to a defect in myogenesis. These data are in agreement with previously reported studies [9], [10], [20].

By the NGS approach we derived that FSHD myogenesis is characterized by a profound dysregulation of miRNA expression showing the involvement of at least 38 known miRNAs, including the myomiRs and possibly three novel miRNAs, but excluding small RNAs previously reported to derive from the D4Z4 array [14]. This and previous works have clearly demonstrated that FSHD cells are characterized by a global dysregulation of mRNA, miRNA and protein expression essentially affecting the myogenic process [9], [10], [11], [21], [24], [44].

The up-regulation of the last *DUX4* gene in individual showing a reduced numbers (≤8) of D4Z4 repeats at 4q35 combined with a specific molecular signature (4A(159/161/168) DUX4 polyadenylation signal (PA) haplotype) is supposed to underlie FSHD pathophysiology [6]. However, it has been recently reported that 1.3% of healthy individuals carry the same molecular signature and 19% of subjects affected by FSHD do not carry alleles with eight or fewer D4Z4 repeats [8]. Furthermore, a dysregulation of genes involved in myogenesis has been recently observed in FSHD fetuses; importantly, the DUX4-fl pathogenic transcript was detected in both FSHD and control samples [45], as well as in unaffected individuals, but not in all FSHD cases [8]. These data suggest that the molecular basis of FSHD might not be simply based on the overexpression of the single *DUX4* gene, but rather from a cascade of dysregulation mediated by the D4Z4 array contraction. This structural alteration, as previously shown, might induce conformational changes in the 4q35 region itself, and perhaps elsewhere in the human genome [46], [47]. Furthermore, in the dysregulation cascade could also play a role lncRNAs, such as DBE-T [7].

## Conclusions

By using the NGS approach, we derived the complete pattern of miRNAs regulating *in vitro* control and FSHD myogenesis. In addition to confirming previously reported FSHD-related miRNAs, we identified additional known and novel miRNAs that are differentially expressed between FSHD and control myogenesis and thus potentially contributing to the FSHD pathogenic mechanism. In general, the comparison of control and FSHD myogenesis reveals that the dystrophy is characterized by a complex alteration of miRNA expression, which also includes the significant down-regulation of myomiRs at late stages of differentiation, thus essentially affecting muscle differentiation and development.

Thus, the full range of molecular alteration(s) at the basis of FSHD is not yet fully deciphered and the miRNA profiling we derive could represent a novel molecular signature for FSHD that includes diagnostic biomarkers and possibly therapeutic targets.

## Materials and Methods

### Cell lines

Primary FSHD and control cell lines were obtained from Myobank-AFM (Institut de Myologie-Groupe Hospitalier Pitié-Salpetrière, Paris) and Boston Biomedical Research Institute (BBRI, Senator Paul D. Wellstone Muscular Dystrophy Cooperative, Research Center for FSHD). Six cell lines derived from biopsies of different healthy and FSHD muscles including vastus, tensor fascia lata, quadriceps femoris (controls) and ilio-psoas and rhomboid (FSHD) (Table S1) were used for deep small RNA sequencing. In addition, to these cell lines, five control and four FSHD cell lines from deltoid and biceps [48] (Table S1) were used to validate deep sequencing data by qRT-PCR. FSHD primary cell lines were derived from biopsies of mild or not affected muscles and showed a D4Z4 array contraction ranging from 5.9 to 28 kb as determined by Southern Blot after EcoRI/BnlI digestion. The results reported below were derived by the analysis of all the cell lines listed in Table S1, comprising nine controls and seven FSHD and thus including also the cells used for NGS. Control and FSHD myoblasts were at low population doubling (from 2 to 7) and highly comparable for the expression of the muscular marker Desmin (96–97%) and the proliferation marker Ki67 (62–65%), as determined by immunofluorescence (Fig. S1). Furthermore, control and FSHD cell lines showed a comparable extent of differentiation as demonstrated by the down-regulation of the proliferation marker Ki67 (by immunofluorescence) and of MYF5 (by qRT-PCR), and by the up-regulation of MYOG (by qRT-PCR), MYOD (by Western blot) and MHC (by qRT-PCR and Western blot), as well as a comparable extent of fusion index (40–45%) (Fig. S1). In addition, FSHD and control myoblasts and myotubes appeared similar when analyzed by immunofluorescence. The cell lines used for NGS originated results in the average comparable to those shown in Fig. S1. Cells were cultured as described in guidelines of BBRI and Cheli et al [9].

### Immunofluorescence, image acquisition and analysis

Cell immunofluorescence was performed as described [49], with antibodies specific for Desmin (rAb, Sigma Aldrich), ki67 (rAb, Vector) and sarcomeric myosin MHC (MF20, from Developmental Studies Hybridoma Bank). Appropriate secondary antibodies conjugated with Alexa 488 (green, Cell Signalling) or Alexa 568 (red; Cell Signalling) were used for fluorescence detection, Nuclei were stained with Hoechst Stain Solution (H6024, SIGMA).

Fluorescent images were taken on confocal laser scanning microscope (Zeiss Lsm 01, Biorad mrc 600, Biorad 1024) using 12× magnification. Images showing double or triple fluorescence were separately acquired using appropriate filters, and the different layers were merged with ImageJ software.

For all control and FSHD cell lines used in this study, the quantification of Desmin and ki67 positive cells has been performed on myoblasts and myotubes. Furthermore, for all control and FSHD cell lines, the absolute fusion index has been calculated as the percentage of MHC-positive nuclei over total number of nuclei after 7 days in differentiation medium.

An average value was determined by counting cells (200–300 cells/field) in at least 5 microscopic fields per sample at 12× magnification.

### RNA isolation and deep sequencing

Total RNA was isolated with the mirVana miRNA isolation kit (cat.# AM1560, Life Technologies) from myoblast cell lines derived from 3 FSHD patients and 3 control subjects, before and after *in vitro* differentiation. RNA was quantified by Nanodrop spectrophotometer (Thermo Scientific) and its integrity was evaluated on an Experion automated electrophoresis system (Bio-Rad); all samples had a RNA Quality Indicator (RQI) value ≥9.

20 micrograms of total RNA were used for PAGE purification of small RNA molecules shorter than 35 nucleotides, adaptor ligation, and small RNA library preparation. The obtained libraries were sequenced on a HiSeq 2000 platform (Illumina) at BGI, Hong Kong, giving approximately 12 million high quality reads per sample (submitted to SRA database under acc. number SRP034654).

### Sequencing data analysis

MicroRNA differential expression analysis was performed using R/Bioconductor, by following the workflow implemented in the oneChannelGUI interface [50], [51]. Briefly, adaptor sequences were trimmed from fastq files using a specific perl script, and then sequences were aligned to the reference human miRBase v.20 precursor dataset (www.mirbase.org) using bowtie 1.0.0. Data were filtered for count threshold (>8 reads in 50% of samples analyzed) and pairwise comparisons of differential miRNA expression were performed using DEseq (log_2_FC≧2; p-value<0.05). Hierarchical clustering of differentially expressed miRNA was performed with dChip (version 2010.01; https://sites.google.com/site/dchipsoft/).

### Identification of novel miRNAs

After excluding all reads that matched known small RNA classes annotated in miRBase v.20 (known miRNAs) and Rfam (e.g. tRNA, snRNA, snoRNA), putative novel miRNAs were predicted using mireap (http://sourceforge.net/projects/mireap/). The program predicts novel miRNAs from deep sequenced small RNA libraries by taking into consideration miRNA biogenesis, sequencing depth, and structural features (hairpin structure and stability) to improve the sensitivity and specificity of miRNA identification. Among predicted novel miRNAs, we considered as plausible candidates those matching the following criteria: 1) the detection in several samples (at least 2 out of 3 samples of one or more experimental groups); 2) the mature miRNA had sufficient sequence support (at least a mean of 10 reads for each experimental group); 3) the sequence did not match to known miRNAs in miRBase v.20.

### Quantitative Real-time PCR

Quantitative RT-PCR (qRT-PCR) analysis was performed on 7900 HT Fast Real-Time PCR System (Applied Biosystems) by TaqMan small RNA Assays to validate the miRNA sequencing data. The miRNA specific probes were from Applied Biosystems. 150 ng RNA was reverse transcribed by TaqMan MicroRNA Reverse Transcription Kit (cat.# 4366596; Applied Biosystems) at 16°C for 30 min, 42°C for 30 min and 85°C for 5 min. Each amplicon was analyzed in duplicate in 96-well plates. TaqMan small RNA Assays reactions were performed following manufacturer's protocol (cat.# 4440048; Applied Biosystems). RNU48 was used for normalization. Thermal cycling conditions for real time PCR were 2 min at 95°C, followed by 40 cycles at 95°C for 10 s and 60°C for 30 s. Results were analyzed using the comparative 2^−ΔΔCt^ method. qRT-PCR experiments for MYF5, MYOG, MHC and GAPDH gene expression analysis were performed as described [9]. The statistical analysis was performed using a two-tail unpaired t-test and the error bars on the graphs are referred to standard deviation. qRT-PCR probes and primers are listed in Table S2.

### Derivation of target genes

The putative miRNAs target genes were predicted by TargetScan Human (http://www.targetscan.org/) [52]. The prediction tool is based on different parameters such as complementarity to the seed region, 3′ complementarity, local AU content, position contribution and conservation in different species [22]. Predicted target genes were then filtered on the basis of their inverse correlation with the expression of mRNAs of two different chip analysis on Affymetrix human exon 1.0 ST array [9], [10], using a FC≥1.5 and a p-value<0.05.

### Pathway and functional annotation analysis

The derived predicted target genes, inversely correlated to the miRNAs expression, were subjected to the analysis of Gene Ontology terms (biological processes) by DAVID (Database for Annotation, Visualization and Integrated Discovery, v6.7) [53], [54]. The target genes were mapped to the GO annotation dataset, and the enriched biological processes were extracted using the EASE score, a modified Fisher exact p-value.

### Protein extracts and Immunoblot analysis

Cells were collected in RIPA Buffer (50 mM TrisHCl pH = 7,4, 150 mM NaCl, 0,1% SDS, 0,5% Deoxycholate Sodium, 1% NP-40 and protease inhibitor cocktail 1X-cat.# P2714-1BTL, Sigma MO, USA), and centrifuged 15 minutes at 13000 rpm at 4°C to discard cellular debris. Sample preparation and Western blot analyses were performed as described in Pisconti et al [55]. After electrophoresis, polypeptides were electrophoretically transferred to nitrocellulose filters (Thermo Scientific) and antigens revealed by the respective primary Abs and the appropriate secondary Abs, through autoradiography using enhanced chemiluminescence (LiteAblot Plus, cat.# EMP011005, Euroclone). In Western blot analyses, primary antibodies against MHC (MF20, from Developmental Studies Hybridoma Bank), MYOD (cat.# sc-31942, Santa Cruz) and housekeeping gene GAPDH (cat.# G8795; Sigma) were used.

## Supporting Information

Figure S1 Characterization of control and FSHD myoblasts cell lines. A) Example of immunostaining experiment on proliferating and differentiated primary myoblasts (control: MX01010MBS; FSHD: MX04309MBS). Images have been taken at confocal laser scanning microscope at 12× magnification. Nuclei were stained with Hoescht (blue). Panels I–IV show localization of Desmin and Ki67 in proliferating myoblasts; panels I–II show immunostaining experiment using the polyclonal anti-Desmin (red); panels II–IV show immunostaining experiment using the polyclonal anti-Ki67 (red). Panels V–VIII show co-localization of Desmin or Ki67 and MHC on differentiated primary myoblasts: panels V–VI show immunostaining with polyclonal anti-Desmin and monoclonal anti-MHC (Ab-Desmin-red and Ab-MHC-green); panels VII–VIII show immunostaining with polyclonal anti-Ki67 and monoclonal anti-MHC (Ab-Ki67-red and Ab-MHC-green). Scale bar = 100 µm. B) Percentage of Desmin and Ki67 positive cells in myoblasts and myotubes after 7 days of differentiation derived from immunostaining with appropriate antibodies (Ab-Desmin and Ab-Ki67). Results are expressed as mean±SD of independent experiments performed on all cell lines described in Table S1. C) Absolute fusion index was determined at day 7 of differentiation (D7), counting the percentage of MHC- positive nuclei over the total number of nuclei. An average value was determined by counting cells in at least 5 microscopic fields (200–300 cells/field). Results are expressed as mean±SD of independent experiments performed on all cell lines (see Table S1). *p<0.05. D) Myogenic differentiation was evaluated by qRT-PCR analysis for MYF5, MYOG, MHC expression. All data points were calculated in triplicate as gene expression relative to endogenous GAPDH expression. Data are represented as the mean±SD of independent experiments performed on all cell lines described in Table S1. GM: growth medium; 7D: seven days of differentiation. *p<0.05, **p<0.01. E) Example of Western blot analysis with specific antibodies against MYOD and MHC in control and FSHD myoblasts at different time points during myogenic differentiation (GM: growth medium; 3D: three days of differentiation; 7D: seven days of differentiation). GAPDH protein level was used as an internal loading control. Graphs show mean values ±SD obtained from the ratio of densitometric values of protein/GAPDH bands. Data are representative of independent experiments performed on all cell lines described in Table S1. The Western blot in E shows a representative experiment (control: MX01010MBS; FSHD: MX04309MBS). *p<0.05, **p<0.01.
(TIF)

Figure S2 Scatter plots of the reads of miRNAs modulated in control and FSHD myogenesis. C1: MX01010MBS; C2: MX03609MBS; C3: MX01110MBS, Control cell lines; F1:MX00409MBS; F2: MX03010MBS; F3:MX04309MBS, FSHD cell lines (see Table S1).
(PDF)

Figure S3 Authentication of NGS data by qRT-PCR. qRT-PCR analysis of myomiRs (miR-1, miR-133a and miR-206) during control and FSHD myogenesis at 0 and 7 days of differentiation on the three control and three FSHD cell lines used in the NGS experiment (MX01010MBS; MX03609MBS; MX01110MBS, MX00409MBS; MX03010MBS; MX04309MBS). GM: growth medium; 7D: seven days of differentiation. *p<0.05; **p<0.01.
(TIF)

Table S1 Primary myoblasts cell lines used in this study. Cell lines have been obtained from Myobank-AFM Istitut de Myologie (Paris)*and Boston Biomedical Research Institute (BBRI, Boston).
(XLSX)

Table S2 Taqman probes and primers used in qRT-PCR experiments.
(XLSX)

Table S3 List of microRNAs modulated in control myogenesis resulting by DEseq analysis.
(XLSX)

Table S4 List of microRNAs modulated in FSHD myogenesis resulting by DEseq analysis.
(XLSX)

Table S5 List of microRNAs modulated in FSHD vs control myotubes resulting by DEseq analysis.
(XLSX)

Table S6 Potentially “validated” targets. List of predicted target genes of miRNAs modulated in control myogenesis, filtered on GSE26061 [9] and GSE26145 [10].
(XLSX)

Table S7 Potentially “validated” targets. List of predicted target genes of miRNAs modulated in FSHD myogenesis, filtered on GSE26061 [9] and GSE26145 [10].
(XLSX)

Table S8 Novel miRNAs predicted by mireap.
(XLSX)
