# Supplementary figures and images for: Next-Generation Sequencing Analysis of MiRNA Expression in Control and FSHD Myogenesis
Source: PLoS One. 2014 Oct 6;9(10):e108411. doi: 10.1371/journal.pone.0108411 (PMC4186784; doi:10.1371/journal.pone.0108411)

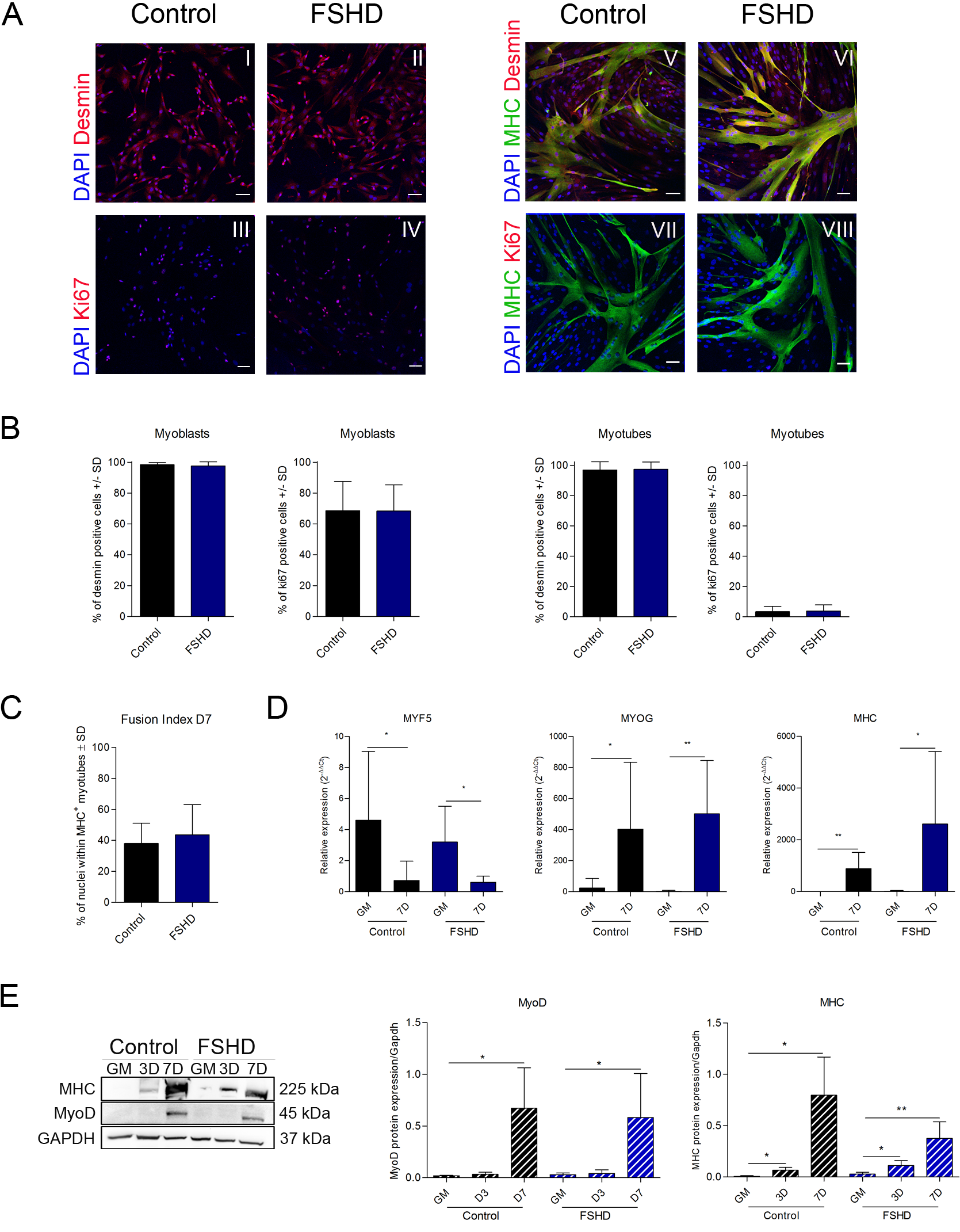

Supplement: Figure S1 — Characterization of control and FSHD myoblasts cell lines. A) Example of immunostaining experiment on proliferating and differentiated primary myoblasts (control: MX01010MBS; FSHD: MX04309MBS). Images have been taken at confocal laser scanning microscope at 12× magnification. Nuclei were stained with Hoescht (blue). Panels I–IV show localization of Desmin and Ki67 in proliferating myoblasts; panels I–II show immunostaining experiment using the polyclonal anti-Desmin (red); panels II–IV show immunostaining experiment using the polyclonal anti-Ki67 (red). Panels V–VIII show co-localization of Desmin or Ki67 and MHC on differentiated primary myoblasts: panels V–VI show immunostaining with polyclonal anti-Desmin and monoclonal anti-MHC (Ab-Desmin-red and Ab-MHC-green); panels VII–VIII show immunostaining with polyclonal anti-Ki67 and monoclonal anti-MHC (Ab-Ki67-red and Ab-MHC-green). Scale bar = 100 µm. B) Percentage of Desmin and Ki67 positive cells in myoblasts and myotubes after 7 days of differentiation derived from immunostaining with appropriate antibodies (Ab-Desmin and Ab-Ki67). Results are expressed as mean±SD of independent experiments performed on all cell lines described in Table S1. C) Absolute fusion index was determined at day 7 of differentiation (D7), counting the percentage of MHC- positive nuclei over the total number of nuclei. An average value was determined by counting cells in at least 5 microscopic fields (200–300 cells/field). Results are expressed as mean±SD of independent experiments performed on all cell lines (see Table S1). *p<0.05. D) Myogenic differentiation was evaluated by qRT-PCR analysis for MYF5, MYOG, MHC expression. All data points were calculated in triplicate as gene expression relative to endogenous GAPDH expression. Data are represented as the mean±SD of independent experiments performed on all cell lines described in Table S1. GM: growth medium; 7D: seven days of differentiation. *p<0.05, **p<0.01. E) Example of Western blot [file pone.0108411.s001.tif]

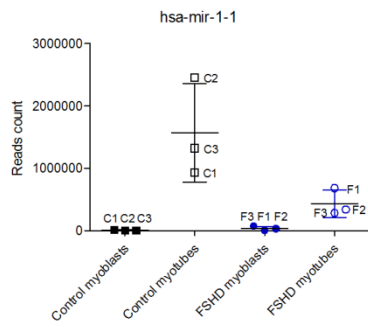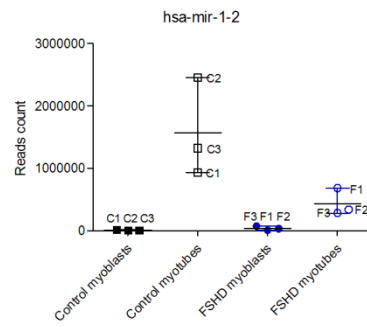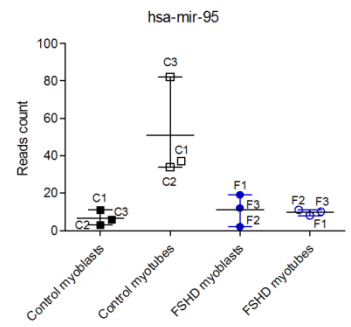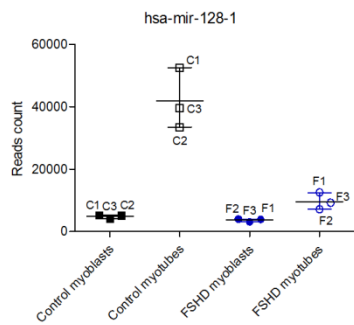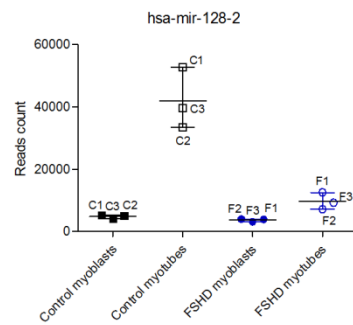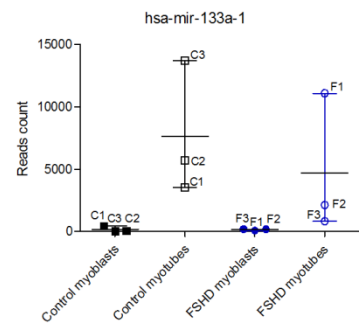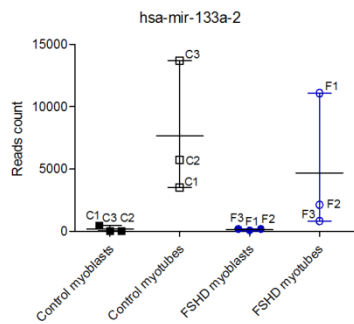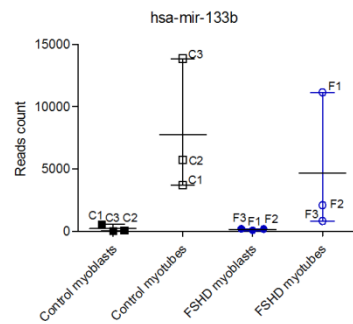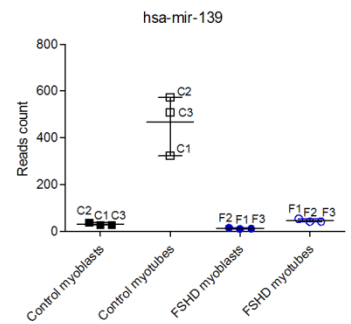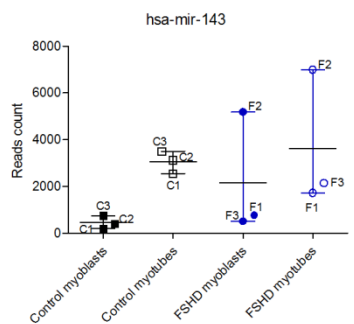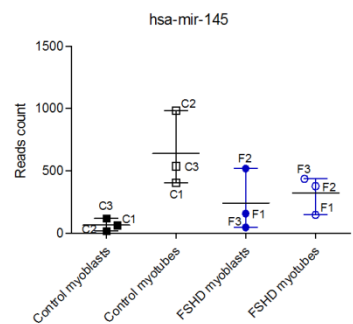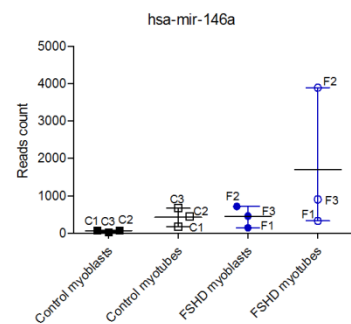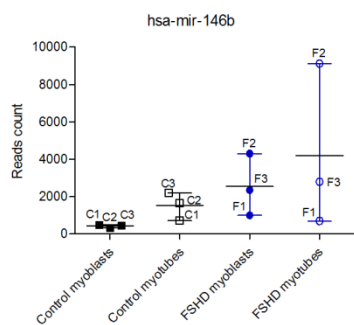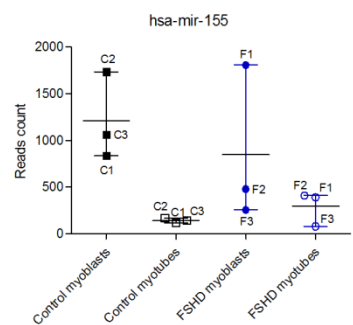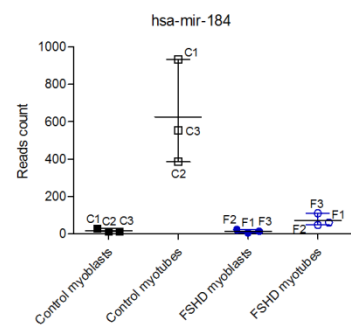

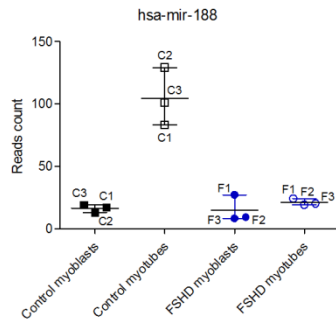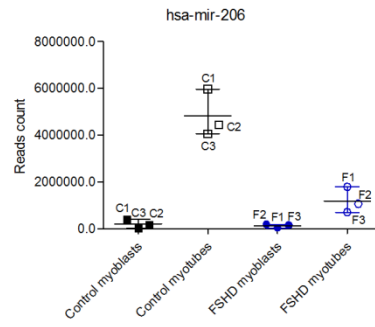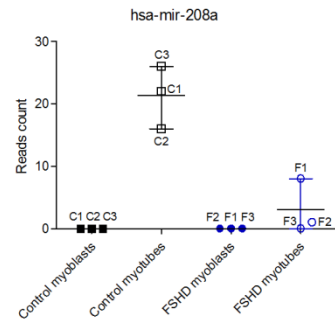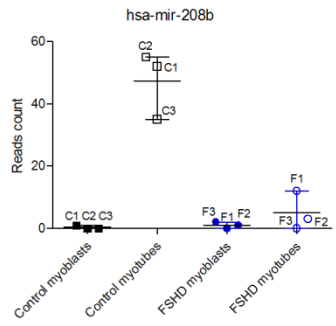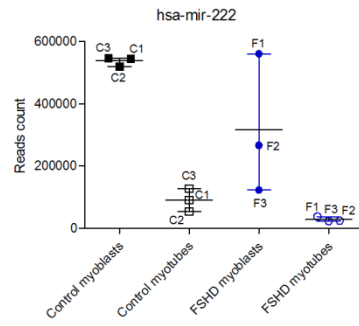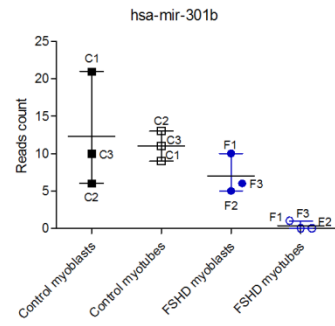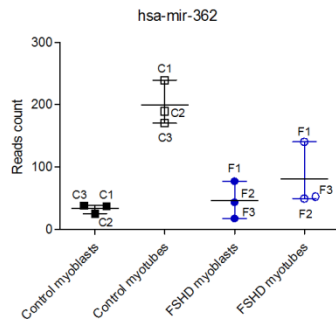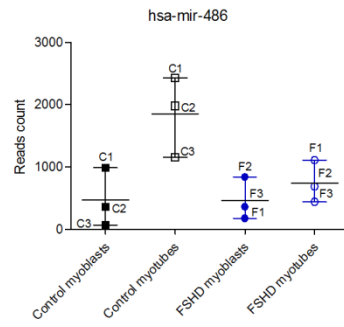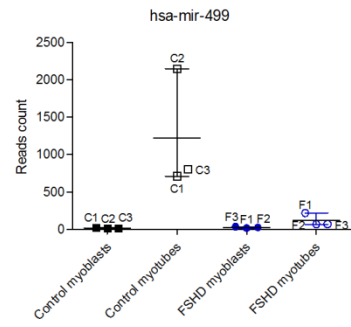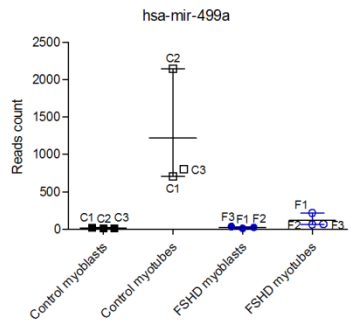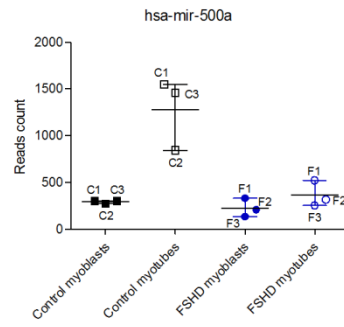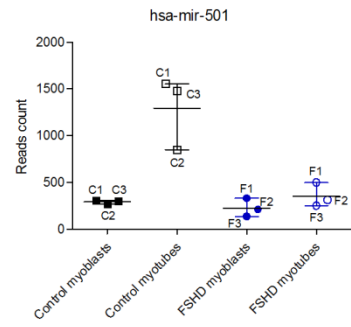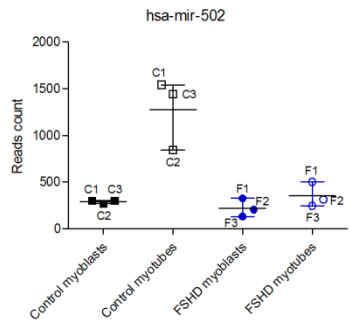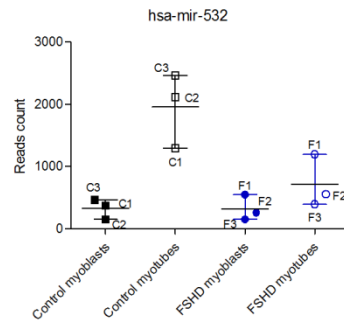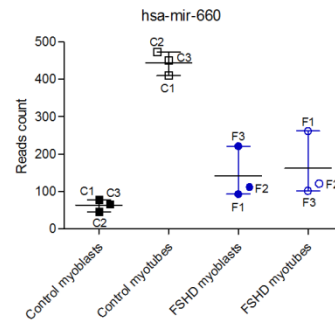

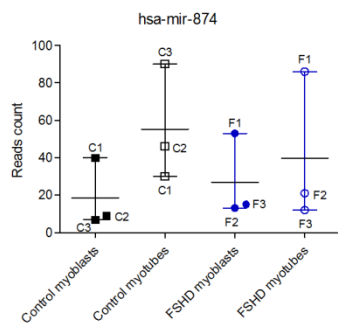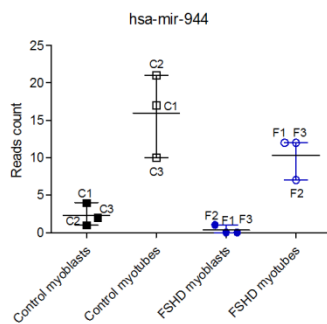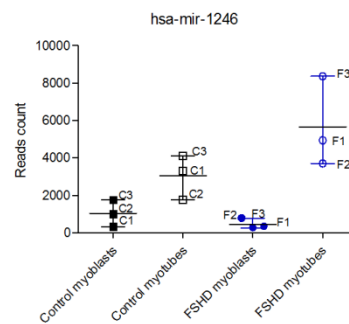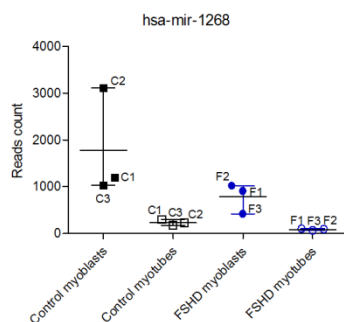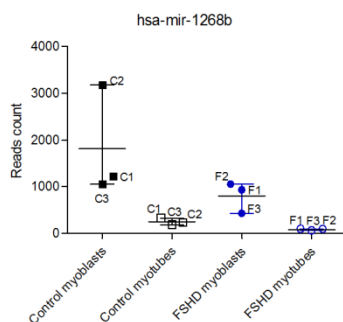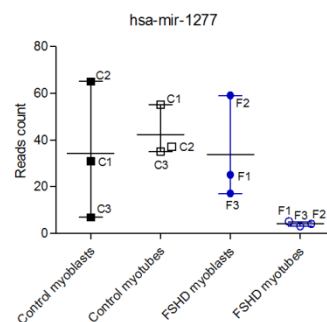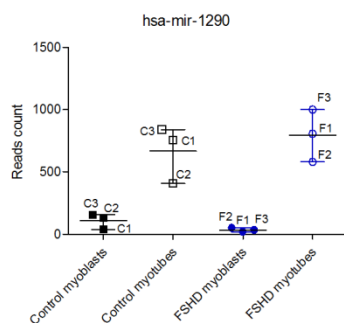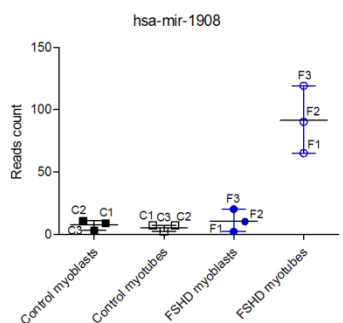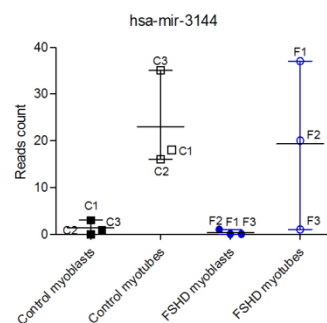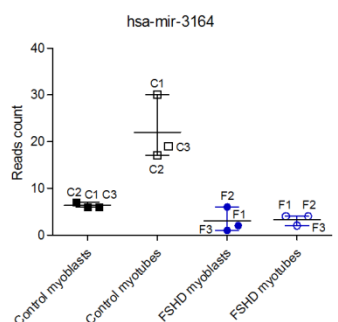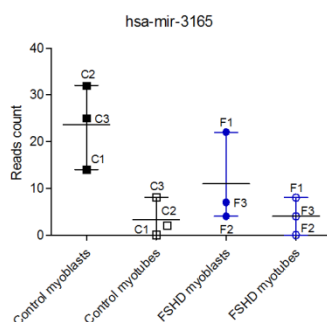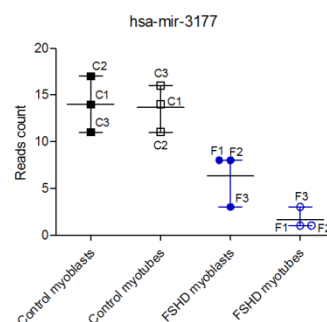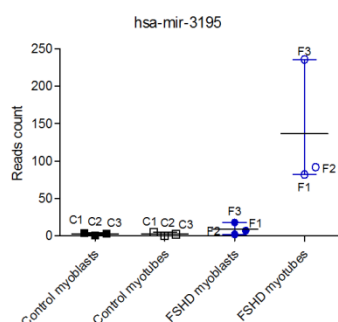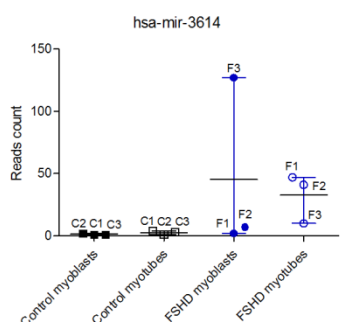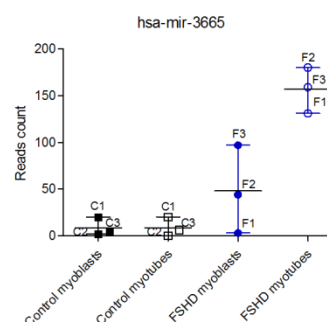

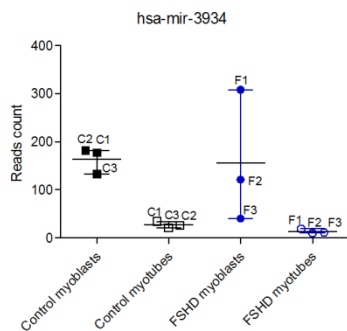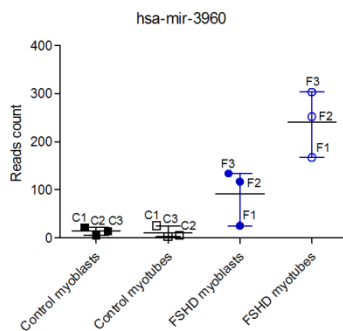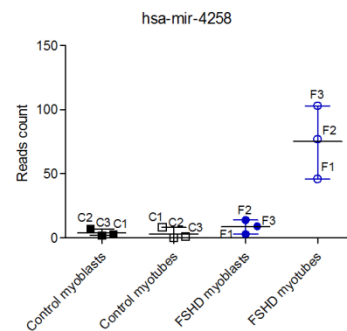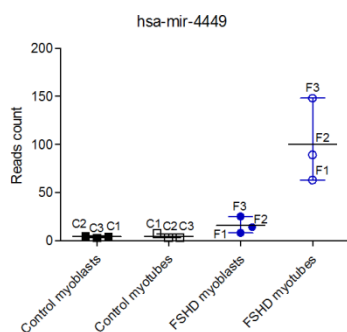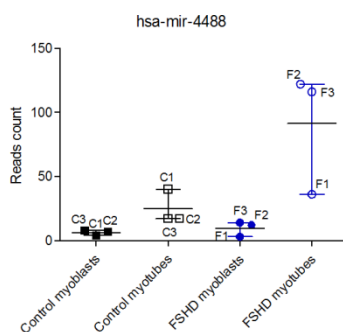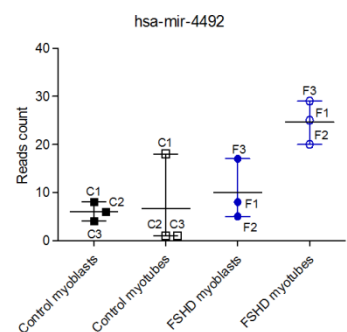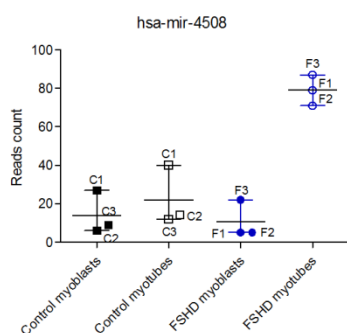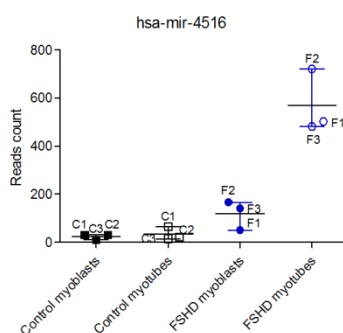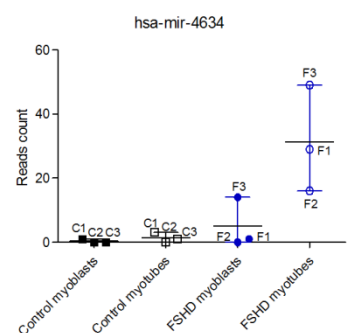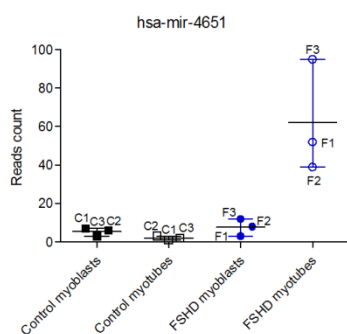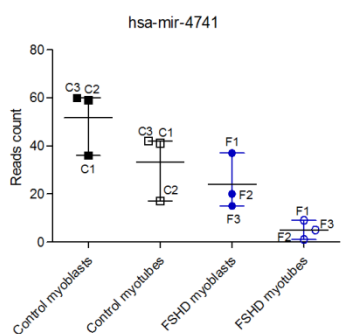

Supplement: Figure S2 — Scatter plots of the reads of miRNAs modulated in control and FSHD myogenesis. C1: MX01010MBS; C2: MX03609MBS; C3: MX01110MBS, Control cell lines; F1:MX00409MBS; F2: MX03010MBS; F3:MX04309MBS, FSHD cell lines (see Table S1). (PDF) [file pone.0108411.s002.pdf]

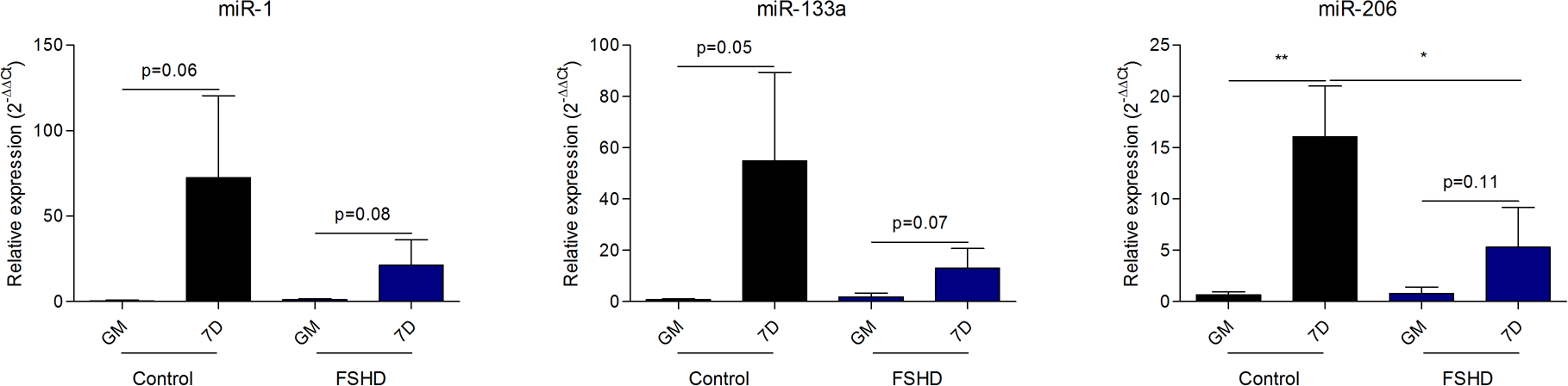

Supplement: Figure S3 — Authentication of NGS data by qRT-PCR. qRT-PCR analysis of myomiRs (miR-1, miR-133a and miR-206) during control and FSHD myogenesis at 0 and 7 days of differentiation on the three control and three FSHD cell lines used in the NGS experiment (MX01010MBS; MX03609MBS; MX01110MBS, MX00409MBS; MX03010MBS; MX04309MBS). GM: growth medium; 7D: seven days of differentiation. *p<0.05; **p<0.01. (TIF) [file pone.0108411.s003.tif]
